# 2-Amino-4,6-dimethyl­pyrimidin-1-ium 1-oxo-2,6,7-trioxa-1λ^5^-phosphabicyclo­[2.2.2]octane-4-carboxyl­ate

**DOI:** 10.1107/S1600536810019896

**Published:** 2010-06-05

**Authors:** Xu-Feng Hou, Gong-Chun Li, Peng-Yang Lai

**Affiliations:** a College of Chemistry and Chemical Engineering, Xuchang University, Xuchang, Henan Province 461000, People’s Republic of China

## Abstract

In the title compound, C_6_H_10_N_3_^+^·C_5_H_6_O_6_P^−^, the cation and anion are linked by pairs of N—H⋯O hydrogen bonds. There are additional inter­molecular N—H⋯N hydrogen bonds, which generate centrosymmetric tetramers of two cations and two anions.

## Related literature

For the applications of caged bicyclic phosphates, see: Li *et al.* (2000 ▶). For related structures, see: Meng *et al.* (2009 ▶); Guo & Zang (2008 ▶); Thakur & Desiraju (2008 ▶); Wang *et al.* (2007 ▶).
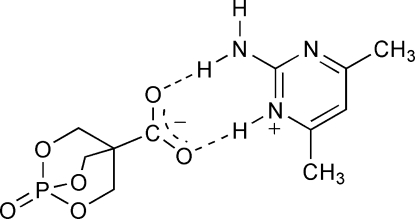


## Experimental

### 

#### Crystal data

C_6_H_10_N_3_^+^·C_5_H_6_O_6_P^−^
*M* *_r_* = 317.24
Monoclinic, 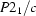

*a* = 9.5080 (13) Å
*b* = 6.1870 (8) Å
*c* = 23.974 (2) Å
β = 99.611 (5)°
*V* = 1390.5 (3) Å^3^
*Z* = 4
Mo *K*α radiation
μ = 0.23 mm^−1^
*T* = 113 K
0.24 × 0.22 × 0.14 mm

#### Data collection

Rigaku Saturn724 CCD diffractometer
Absorption correction: multi-scan (*CrystalClear*; Rigaku/MSC, 2009 ▶) *T*_min_ = 0.947, *T*_max_ = 0.969
23989 measured reflections
6532 independent reflections
4883 reflections with *I* > 2σ(*I*)
*R*_int_ = 0.035

#### Refinement

*R*[*F* ^2^ > 2σ(*F* ^2^)] = 0.039
*wR*(*F* ^2^) = 0.107
*S* = 1.01
6532 reflections
204 parameters
H atoms treated by a mixture of independent and constrained refinement
Δρ_max_ = 0.54 e Å^−3^
Δρ_min_ = −0.38 e Å^−3^

### 

Data collection: *CrystalClear-SM Expert* (Rigaku/MSC, 2009 ▶); cell refinement: *CrystalClear-SM Expert*; data reduction: *CrystalClear-SM Expert*; program(s) used to solve structure: *SHELXS97* (Sheldrick, 2008 ▶); program(s) used to refine structure: *SHELXL97* (Sheldrick, 2008 ▶); molecular graphics: *CrystalStructure* (Rigaku/MSC, 2009 ▶); software used to prepare material for publication: *CrystalStructure*.

## Supplementary Material

Crystal structure: contains datablocks global, I. DOI: 10.1107/S1600536810019896/fj2308sup1.cif

Structure factors: contains datablocks I. DOI: 10.1107/S1600536810019896/fj2308Isup2.hkl

Additional supplementary materials:  crystallographic information; 3D view; checkCIF report

## Figures and Tables

**Table 1:** Hydrogen-bond geometry (Å, °)

*D*—H⋯*A*	*D*—H	H⋯*A*	*D*⋯*A*	*D*—H⋯*A*
N1—H1⋯O1	0.866 (15)	1.907 (15)	2.7719 (11)	176.4 (14)
N1—H2⋯N3^i^	0.900 (14)	2.113 (15)	3.0114 (12)	176.3 (12)
N2—H3⋯O2	0.988 (15)	1.738 (16)	2.7170 (10)	170.4 (15)

Symmetry code: (i) 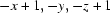.

**Fig. 1.**
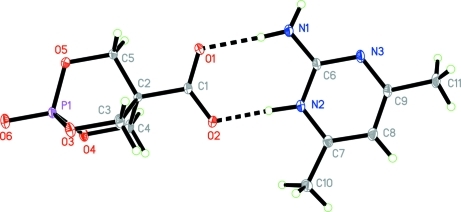
The asymmetric unit of the title compound, (I), with displacement ellipsoids drawn at the 30% probability level.

**Fig. 2.**
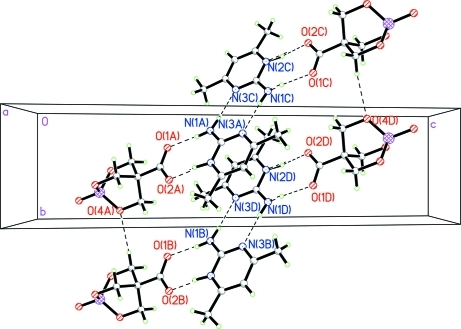
The packing diagram of the title compound. Intermolecular hydrogen bonds are shown as dashed line.

## supplementary crystallographic information

### Comment

Caged bicyclic phosphates are widely used as flame retardants or pesticides (Li *et al.* 2000). It can also serve as host–guest systems and have been studied in the context of hydrogen-bond patterns (Guo & Zang, 2008; Wang *et al.*, 2007). Aminopyrimidine derivatives are biologically important compounds as they occur in nature as components of nucleic acids (Meng *et al.*, 2009). The crystal structures of aminopyrimidine carboxylates have been reported (Thakur *et al.*, 2008). We report here the crystal structure of a new bicyclic phosphate cage compound.

In the title compound, [C_6_H_10_N_3_]^+^ [C_5_H_6_O_6_P]^-^, The cation and anion in the asymmetric unit are linked by N—H···O hydrogen bonds. There is also addition intermolecular N—H···N hydrogen bonds. (Fig. 2).

### Experimental

The title compound was obtained by reaction of 1-oxo-2,6,7-trioxa-1-phosphabicyclo[2.2.2]octane-4-carboxylic acid (0.39 g, 0.2 mmol) and 2-amino-4,6-dimethylpyrimidine(0.25 g, 0.2 mmol) in refluxing acetone (50 ml). The solvent was evaporated *in vacuo*. The title compound was recrystallized from ethanol and single crystals of (I) were obtained by slow evaporation.

### Refinement

All H atoms were placed in calculated positions, with C—H = 0.98 Å or 0.99 Å, and included in the final cycles of refinement using a riding model, with *U*_iso_(H) = 1.2Ueq(C).

### Crystal data

**Table:**

C_6_H_10_N_3_^+^·C_5_H_6_O_6_P^−^	*F*(000) = 664
*M**_r_* = 317.24	*D*_x_ = 1.515 Mg m^−^^3^
Monoclinic, *P*2_1_/*c*	Mo *K*α radiation, λ = 0.71075 Å
Hall symbol: -P 2ybc	Cell parameters from 5985 reflections
*a* = 9.5080 (13) Å	θ = 2.2–36.3°
*b* = 6.1870 (8) Å	µ = 0.23 mm^−^^1^
*c* = 23.974 (2) Å	*T* = 113 K
β = 99.611 (5)°	Prism, colorless
*V* = 1390.5 (3) Å^3^	0.24 × 0.22 × 0.14 mm
*Z* = 4	

### Data collection

**Table:**

Rigaku Saturn724 CCD diffractometer	6532 independent reflections
Radiation source: rotating anode	4883 reflections with *I* > 2σ(*I*)
multilayer	*R*_int_ = 0.035
Detector resolution: 14.222 pixels mm^-1^	θ_max_ = 36.5°, θ_min_ = 1.7°
ω scans	*h* = −15→14
Absorption correction: multi-scan (*CrystalClear*; Rigaku/MSC, 2009)	*k* = −10→10
*T*_min_ = 0.947, *T*_max_ = 0.969	*l* = −36→39
23989 measured reflections	

### Refinement

**Table:**

Refinement on *F*^2^	Primary atom site location: structure-invariant direct methods
Least-squares matrix: full	Secondary atom site location: difference Fourier map
*R*[*F*^2^ > 2σ(*F*^2^)] = 0.039	Hydrogen site location: inferred from neighbouring sites
*wR*(*F*^2^) = 0.107	H atoms treated by a mixture of independent and constrained refinement
*S* = 1.01	*w* = 1/[σ^2^(*F*_o_^2^) + (0.0615*P*)^2^] where *P* = (*F*_o_^2^ + 2*F*_c_^2^)/3
6532 reflections	(Δ/σ)_max_ = 0.001
204 parameters	Δρ_max_ = 0.54 e Å^−^^3^
0 restraints	Δρ_min_ = −0.38 e Å^−^^3^

### Special details

**Table:**

Geometry. All e.s.d.'s (except the e.s.d. in the dihedral angle between two l.s. planes) are estimated using the full covariance matrix. The cell e.s.d.'s are taken into account individually in the estimation of e.s.d.'s in distances, angles and torsion angles; correlations between e.s.d.'s in cell parameters are only used when they are defined by crystal symmetry. An approximate (isotropic) treatment of cell e.s.d.'s is used for estimating e.s.d.'s involving l.s. planes.
Refinement. Refinement of *F*^2^ against ALL reflections. The weighted *R*-factor *wR* and goodness of fit *S* are based on *F*^2^, conventional *R*-factors *R* are based on *F*, with *F* set to zero for negative *F*^2^. The threshold expression of *F*^2^ > σ(*F*^2^) is used only for calculating *R*-factors(gt) *etc*. and is not relevant to the choice of reflections for refinement. *R*-factors based on *F*^2^ are statistically about twice as large as those based on *F*, and *R*- factors based on ALL data will be even larger.

### Fractional atomic coordinates and isotropic or equivalent isotropic displacement parameters (Å^2^)

**Table:**

	*x*	*y*	*z*	*U*_iso_*/*U*_eq_	
P1	0.14090 (2)	0.74372 (4)	0.157866 (9)	0.01584 (6)	
O1	0.32415 (8)	0.33013 (12)	0.32735 (3)	0.02813 (17)	
O2	0.46621 (8)	0.62093 (12)	0.33500 (3)	0.02544 (16)	
O3	0.29862 (7)	0.67033 (12)	0.15596 (3)	0.02097 (14)	
O4	0.15812 (7)	0.91396 (11)	0.20765 (3)	0.02023 (13)	
O5	0.06923 (7)	0.54214 (12)	0.18168 (3)	0.02421 (15)	
O6	0.06557 (8)	0.82445 (13)	0.10419 (3)	0.02619 (16)	
N1	0.45629 (9)	0.17785 (15)	0.43177 (4)	0.02312 (17)	
N2	0.61028 (8)	0.46847 (12)	0.43466 (3)	0.01702 (14)	
N3	0.63811 (8)	0.21010 (14)	0.50821 (3)	0.01949 (15)	
C1	0.36366 (9)	0.50774 (14)	0.31115 (4)	0.01674 (15)	
C2	0.27993 (8)	0.59650 (13)	0.25498 (3)	0.01332 (14)	
C3	0.37600 (9)	0.58891 (16)	0.20974 (4)	0.01990 (17)	
H3A	0.4621	0.6782	0.2219	0.024*	
H3B	0.4070	0.4383	0.2049	0.024*	
C4	0.23414 (10)	0.83103 (15)	0.26188 (4)	0.02018 (17)	
H4A	0.1711	0.8386	0.2908	0.024*	
H4B	0.3193	0.9214	0.2749	0.024*	
C5	0.14789 (10)	0.45860 (16)	0.23533 (4)	0.02254 (19)	
H5A	0.1769	0.3072	0.2302	0.027*	
H5B	0.0854	0.4605	0.2645	0.027*	
C6	0.56840 (9)	0.28450 (15)	0.45830 (4)	0.01698 (15)	
C7	0.72567 (10)	0.58213 (15)	0.45997 (4)	0.01896 (16)	
C8	0.79892 (10)	0.51036 (16)	0.51086 (4)	0.02109 (17)	
H8	0.8792	0.5871	0.5300	0.025*	
C9	0.75166 (10)	0.32090 (16)	0.53356 (4)	0.01951 (17)	
C10	0.76334 (12)	0.77807 (16)	0.42947 (5)	0.0264 (2)	
H10A	0.8040	0.7342	0.3962	0.032*	
H10B	0.8334	0.8642	0.4547	0.032*	
H10C	0.6774	0.8647	0.4173	0.032*	
C11	0.82793 (13)	0.22887 (19)	0.58813 (4)	0.0295 (2)	
H11A	0.7581	0.1869	0.6120	0.035*	
H11B	0.8927	0.3379	0.6079	0.035*	
H11C	0.8828	0.1016	0.5803	0.035*	
H1	0.4144 (16)	0.231 (2)	0.3998 (7)	0.033 (4)*	
H2	0.4287 (15)	0.065 (2)	0.4512 (6)	0.039 (4)*	
H3	0.5623 (16)	0.511 (3)	0.3965 (6)	0.052 (4)*	

### Atomic displacement parameters (Å^2^)

**Table:**

	*U*^11^	*U*^22^	*U*^33^	*U*^12^	*U*^13^	*U*^23^
P1	0.01665 (11)	0.01722 (10)	0.01235 (10)	0.00168 (8)	−0.00133 (7)	0.00215 (7)
O1	0.0314 (4)	0.0245 (3)	0.0237 (3)	−0.0074 (3)	−0.0093 (3)	0.0116 (3)
O2	0.0232 (3)	0.0255 (3)	0.0229 (3)	−0.0060 (3)	−0.0101 (3)	0.0070 (3)
O3	0.0215 (3)	0.0277 (3)	0.0142 (3)	0.0069 (3)	0.0043 (2)	0.0034 (2)
O4	0.0265 (3)	0.0171 (3)	0.0155 (3)	0.0085 (2)	−0.0013 (2)	0.0013 (2)
O5	0.0211 (3)	0.0261 (3)	0.0213 (3)	−0.0082 (3)	−0.0087 (2)	0.0080 (3)
O6	0.0282 (4)	0.0310 (4)	0.0167 (3)	0.0031 (3)	−0.0042 (3)	0.0060 (3)
N1	0.0190 (4)	0.0305 (4)	0.0177 (4)	−0.0039 (3)	−0.0029 (3)	0.0094 (3)
N2	0.0183 (3)	0.0190 (3)	0.0127 (3)	0.0026 (3)	−0.0005 (2)	0.0021 (2)
N3	0.0171 (3)	0.0277 (4)	0.0127 (3)	0.0015 (3)	−0.0002 (3)	0.0054 (3)
C1	0.0161 (4)	0.0180 (4)	0.0149 (4)	0.0020 (3)	−0.0012 (3)	0.0028 (3)
C2	0.0126 (3)	0.0130 (3)	0.0134 (3)	0.0002 (3)	−0.0007 (3)	0.0011 (3)
C3	0.0157 (4)	0.0266 (4)	0.0171 (4)	0.0057 (3)	0.0018 (3)	0.0032 (3)
C4	0.0240 (4)	0.0190 (4)	0.0151 (4)	0.0073 (3)	−0.0040 (3)	−0.0018 (3)
C5	0.0200 (4)	0.0239 (4)	0.0203 (4)	−0.0077 (3)	−0.0065 (3)	0.0090 (3)
C6	0.0151 (4)	0.0231 (4)	0.0125 (3)	0.0028 (3)	0.0016 (3)	0.0034 (3)
C7	0.0219 (4)	0.0185 (4)	0.0157 (4)	0.0021 (3)	0.0009 (3)	−0.0016 (3)
C8	0.0231 (4)	0.0231 (4)	0.0154 (4)	0.0002 (3)	−0.0018 (3)	−0.0015 (3)
C9	0.0187 (4)	0.0273 (4)	0.0116 (3)	0.0031 (3)	−0.0002 (3)	0.0010 (3)
C10	0.0334 (5)	0.0182 (4)	0.0247 (5)	−0.0026 (4)	−0.0033 (4)	0.0018 (3)
C11	0.0280 (5)	0.0417 (6)	0.0151 (4)	−0.0023 (4)	−0.0070 (4)	0.0075 (4)

### Geometric parameters (Å, °)

**Table:**

P1—O6	1.4529 (7)	C2—C3	1.5310 (12)
P1—O5	1.5734 (7)	C2—C4	1.5318 (12)
P1—O3	1.5748 (7)	C3—H3A	0.9900
P1—O4	1.5798 (7)	C3—H3B	0.9900
O1—C1	1.2443 (11)	C4—H4A	0.9900
O2—C1	1.2578 (11)	C4—H4B	0.9900
O3—C3	1.4631 (11)	C5—H5A	0.9900
O4—C4	1.4703 (10)	C5—H5B	0.9900
O5—C5	1.4694 (11)	C7—C8	1.3737 (12)
N1—C6	1.3227 (12)	C7—C10	1.4901 (14)
N1—H1	0.866 (15)	C8—C9	1.3977 (14)
N1—H2	0.900 (14)	C8—H8	0.9500
N2—C7	1.3580 (12)	C9—C11	1.4983 (13)
N2—C6	1.3603 (12)	C10—H10A	0.9800
N2—H3	0.988 (15)	C10—H10B	0.9800
N3—C9	1.3363 (12)	C10—H10C	0.9800
N3—C6	1.3484 (11)	C11—H11A	0.9800
C1—C2	1.5460 (11)	C11—H11B	0.9800
C2—C5	1.5260 (12)	C11—H11C	0.9800
			
O6—P1—O5	114.50 (4)	O4—C4—H4B	109.7
O6—P1—O3	113.86 (4)	C2—C4—H4B	109.7
O5—P1—O3	104.73 (4)	H4A—C4—H4B	108.2
O6—P1—O4	114.43 (4)	O5—C5—C2	110.24 (7)
O5—P1—O4	104.52 (4)	O5—C5—H5A	109.6
O3—P1—O4	103.59 (4)	C2—C5—H5A	109.6
C3—O3—P1	114.52 (5)	O5—C5—H5B	109.6
C4—O4—P1	114.14 (5)	C2—C5—H5B	109.6
C5—O5—P1	114.11 (5)	H5A—C5—H5B	108.1
C6—N1—H1	116.8 (10)	N1—C6—N3	119.58 (8)
C6—N1—H2	114.7 (9)	N1—C6—N2	119.05 (8)
H1—N1—H2	128.2 (13)	N3—C6—N2	121.37 (8)
C7—N2—C6	121.20 (7)	N2—C7—C8	118.78 (8)
C7—N2—H3	119.3 (10)	N2—C7—C10	116.39 (8)
C6—N2—H3	119.1 (10)	C8—C7—C10	124.83 (9)
C9—N3—C6	117.95 (8)	C7—C8—C9	117.94 (8)
O1—C1—O2	126.96 (8)	C7—C8—H8	121.0
O1—C1—C2	116.56 (7)	C9—C8—H8	121.0
O2—C1—C2	116.46 (7)	N3—C9—C8	122.75 (8)
C5—C2—C3	108.75 (7)	N3—C9—C11	116.02 (9)
C5—C2—C4	109.20 (7)	C8—C9—C11	121.23 (9)
C3—C2—C4	108.63 (7)	C7—C10—H10A	109.5
C5—C2—C1	110.36 (7)	C7—C10—H10B	109.5
C3—C2—C1	108.95 (7)	H10A—C10—H10B	109.5
C4—C2—C1	110.90 (7)	C7—C10—H10C	109.5
O3—C3—C2	110.00 (7)	H10A—C10—H10C	109.5
O3—C3—H3A	109.7	H10B—C10—H10C	109.5
C2—C3—H3A	109.7	C9—C11—H11A	109.5
O3—C3—H3B	109.7	C9—C11—H11B	109.5
C2—C3—H3B	109.7	H11A—C11—H11B	109.5
H3A—C3—H3B	108.2	C9—C11—H11C	109.5
O4—C4—C2	109.99 (7)	H11A—C11—H11C	109.5
O4—C4—H4A	109.7	H11B—C11—H11C	109.5
C2—C4—H4A	109.7		
			
O6—P1—O3—C3	179.91 (6)	C5—C2—C4—O4	−59.68 (9)
O5—P1—O3—C3	−54.29 (7)	C3—C2—C4—O4	58.79 (9)
O4—P1—O3—C3	55.00 (7)	C1—C2—C4—O4	178.50 (7)
O6—P1—O4—C4	179.86 (6)	P1—O5—C5—C2	1.39 (11)
O5—P1—O4—C4	53.84 (7)	C3—C2—C5—O5	−60.03 (10)
O3—P1—O4—C4	−55.60 (7)	C4—C2—C5—O5	58.35 (10)
O6—P1—O5—C5	178.68 (7)	C1—C2—C5—O5	−179.49 (7)
O3—P1—O5—C5	53.28 (8)	C9—N3—C6—N1	179.42 (9)
O4—P1—O5—C5	−55.34 (8)	C9—N3—C6—N2	−1.06 (13)
O1—C1—C2—C5	8.87 (11)	C7—N2—C6—N1	−179.24 (9)
O2—C1—C2—C5	−172.46 (9)	C7—N2—C6—N3	1.24 (13)
O1—C1—C2—C3	−110.46 (9)	C6—N2—C7—C8	−1.19 (13)
O2—C1—C2—C3	68.20 (10)	C6—N2—C7—C10	178.60 (8)
O1—C1—C2—C4	130.02 (9)	N2—C7—C8—C9	0.99 (13)
O2—C1—C2—C4	−51.31 (11)	C10—C7—C8—C9	−178.78 (9)
P1—O3—C3—C2	0.12 (9)	C6—N3—C9—C8	0.90 (14)
C5—C2—C3—O3	59.14 (9)	C6—N3—C9—C11	−178.44 (9)
C4—C2—C3—O3	−59.61 (9)	C7—C8—C9—N3	−0.89 (14)
C1—C2—C3—O3	179.47 (7)	C7—C8—C9—C11	178.42 (9)
P1—O4—C4—C2	1.10 (9)		

### Hydrogen-bond geometry (Å, °)

**Table:**

*D*—H···*A*	*D*—H	H···*A*	*D*···*A*	*D*—H···*A*
N1—H1···O1	0.866 (15)	1.907 (15)	2.7719 (11)	176.4 (14)
N1—H2···N3^i^	0.900 (14)	2.113 (15)	3.0114 (12)	176.3 (12)
N2—H3···O2	0.988 (15)	1.738 (16)	2.7170 (10)	170.4 (15)

Symmetry codes: (i) −*x*+1, −*y*, −*z*+1.
